# Value Attribution in the Decision to Use of Whole Body MRI for Early Cancer Diagnosis

**DOI:** 10.3390/diagnostics11060972

**Published:** 2021-05-28

**Authors:** Derna Busacchio, Ketti Mazzocco, Davide Radice, Paul E. Summers, Paola Pricolo, Gabriella Pravettoni, Giuseppe Petralia

**Affiliations:** 1 Applied Research Division for Cognitive and Psychological Science, IEO, European Institute of Oncology IRCCS, Via Ripamonti 435, 20141 Milan, Italy; ketti.mazzocco@ieo.it (K.M.); gabriella.pravettoni@ieo.it (G.P.); 2 Division of Radiology, IEO European Institute of Oncology IRCCS, Via Ripamonti 435, 20141 Milan, Italy; paul.summers@ieo.it (P.E.S.); paola.pricolo@ieo.it (P.P.); 3 Department of Oncology and Hemato-Oncology, University of Milan, 20122 Milan, Italy; giuseppe.petralia@ieo.it; 4 Division of Epidemiology and Biostatistics, IEO European Institute of Oncology IRCCS, Via Ripamonti 435, 20141 Milan, Italy; davide.radice@ieo.it; 5 Precision Imaging and Research Unit, Department of Medical Imaging and Radiation Sciences, IEO European Institute of Oncology IRCCS, Via Ripamonti 435, 20141 Milan, Italy

**Keywords:** diffusion whole body, magnetic resonance, cancer screening, decision aids, preferences

## Abstract

This study aimed to identify the main factors that asymptomatic individuals considered when deciding to undergo self-referred Whole-body MRI (WB-MRI) for early cancer diagnosis and the subjective values attributed to each mentioned factor in a Decision tree analysis. Personal characteristics such as risk perception and personality were investigated as possible factors affecting value attribution. Seventy-four volunteers (mean age 56.4; male = 47) filled a simplified decision tree by expressing the expected factors and related subjective values associated with two screening options for early cancer diagnosis (standard procedures vs. WB-MRI+standard procedures) while waiting for a WB-MRI examination. Questionnaires on risk perception and personality traits were also administered. Expected factors were summarized in 5 clusters: diagnostic certainty, psychological well-being, safety, test validity and time/cost. Test validity and time/cost were evaluated as potential losses in both procedures. Diagnostic Certainty and safety were evaluated as losses in standard screening, and as an advantage when considering WB-MRI+standard screening. Forty-five percent of participants considered WB-MRI+standard screening as beneficial for their psychological well-being. Finally, personal absolute and comparative risk to get cancer was associated with a positive value attribution to WB-MRI (*p* < 0.05). Our results showed the addition of WB-MRI to be generally considered a good option to increase individuals’ perceptions of diagnostic certainty and the safety of the exam, and to increase psychological well-being. The positive value of such a screening option increased with the individual’s cancer risk perception.

## 1. Introduction

Given the growing interest in the adoption of Whole-body MRI (WB-MRI) for early cancer diagnosis, assessing factors that move healthy people to adopt this new technology may help physicians and health institutions to tailor communication to facilitate individuals’ understanding and decision making regarding such screening. WB-MRI is an advanced imaging technique that allows the detection, staging and therapy monitoring of several different cancer histotypes and cancer-prone syndromes [1,2] and several studies have shown its consistent performance in early cancer diagnosis amongst asymptomatic individuals [3]. The lack of radiation exposure and absence of contrast-agent administration in a typical WB-MRI examination, contribute to its attractiveness for early cancer diagnosis, as an adjunct to standard screening examinations. In our country, WB-MRI can be performed at one’s own expense, as an adjunct to standard screening examinations (mammography, pap-test, FOBT and PSA) provided by National Health Service. At an individual level, risk perception can guide protective actions, such as screening for cancer, quitting smoking [4], nutrition habits and physical activity [5,6].

Moreover, the judgments people make about their risk of developing cancer have important implications for cancer prevention, screening and treatment [7,8,9,10].

People who feel a higher risk to get cancer will spend more time seeking out information to reduce the risk [11], also they will be more motivated to follow screening programs [12]. When fear of cancer is overly high, however, it may act as a roadblock to screening decisions due to avoidant or fatalistic thoughts about the risk occurrence [9]. Individual characteristics that have been considered as influencing risk perception [13] and choice include gender, age, education background, income levels [14], and personality factors [15]. The WB-MRI technique has attained a high level of acceptability among both oncological patients and asymptomatic subjects [16,17]. Specifically, acceptability towards WB-MRI seems to depend on the person’s expectations. For example, the level of discomfort expected to be felt by the person during the procedure was stronger than the actual experienced one [17].

At present, however, no studies have investigated the subjective factors that may guide the person’s decision to self-refer to WB-MRI for early cancer diagnosis.

According to Tversky and Kahneman [18,19,20], the mental representations individuals construct of a problem depends on how the problematic situation is structured. Furthermore, the same situation will be evaluated as a gain or a loss based on the individual’s reference point. Consideration of multiple factors makes information processing and consequent decisions a complex and sometimes difficult process.

This is particularly true when dealing with health information: a study revealed that 47% of the general population faced difficulties in understanding, judging and using the information to make decisions regarding their health [21,22], even in the absence of disease. This is an important obstacle to the realization of the concept of an informed decision in medicine. Fully informed decision making, in fact, occurs only when people understand the real benefits and harms of different care options, and can integrate this information with their preferences and needs. Current decision aids (DA) generally present structured information for patients to read prior to making a choice. An adapted form of Decision Tree (DT) construction could help support individuals in the evaluation of options. A DT is a flowchart-like tool used in the decision to choose the option that guarantees the highest subjective expected value through a simple graphic representation of gathered knowledge (values and probabilities) [23]. Given the simplicity of the graphic representation, DTs could be a good tool for supporting patients, or healthy persons in actively evaluating available options and constructing their preferences based on associated advantages, disadvantages and personal expectations. Indeed, it provides the possibility to form a graphic representation of the individual’s values associated with the main factors that contributed to the decision and to order the available information.

The present work is an exploratory, sequential case-series study aimed to identify the main factors that individuals considered when deciding to undergo WB-MRI for early cancer diagnosis, and the subjective values attributed to each mentioned factor in a DT analysis. The values associated with each factor in the DT were used to form a representation of the expected value participants’ associate with the considered procedure.

We further aimed to investigate the influence of personal cancer risk perception and personality characteristics on the evaluation process.

## 2. Materials and Methods

### 2.1. Participant Recruitment and Sampling

Participants were individuals who self-referred to WB-MRI for early cancer diagnosis at the <Institution name blinded for review> between August 2019 and December 2019. Our institution offers WB-MRI to self-referring asymptomatic subjects, who pay out of pocket for the examination, as an adjunct to the Italian National Health Service programmed screening that involves mammography, Papanikolau “Pap test” or faecal occult blood test “FOBT” [24] (herein referred to as standard screening).

Eligible participants were identified and approached while waiting for the WB-MRI examination, and those who agreed to participate provided signed informed consent prior to completing the study questionnaires. The institution’s ethical committee approved the study (Trial ID IEO1032).

Inclusion criteria were being asymptomatic and without contraindication to MRI examination (e.g., pacemaker, pregnancy in the first trimester, metal implants). The participants in our cohort had self-referred for WB-MRI for early cancer diagnosis in addition to their participation in the aforementioned standard screening examinations.

All participants were informed that WB-MRI does not replace screening offered by public health services. The WB-MRI protocol has been previously published [17,25,26].

### 2.2. Measures

Sociodemographic and screening examination history, including gender and age, were collected from participants.

Before the WB-MRI examination, participants were asked to create a simplified Decision Tree to express, in a graphical representation, the main factors they expected to be associated with the two cancer screening options: (1) standard screening tests; (2) standard screening tests plus WB-MRI.

Participants were also asked to complete two questionnaires to assess risk perception and personality traits. Specifically, a questionnaire adapted from Veldhuijzen et al. 2006 [27] was used to examine the patient’s perceptions of their personal and comparative risk to develop cancer in the future and of the severity/seriousness of cancer (rated from 1= very unlikely to 5 = very likely). Participants were asked to indicate how likely (from 1= very unlikely to 5 = very likely) it is that they will develop cancer.

Further, Big Five Inventory-10 (BFI-10) was used to investigate personality dimensions as these may provide salient information on differences in individuals’ decision-making process and attitudes toward WB-MRI. The BFI-10 is composed of 10 items, evaluated on a Likert scale from 1 (strongly disagree) to 5 (strongly agree) [28] that assesses 5 personality dimensions: extraversion, agreeableness, conscientiousness, emotional stability and openness to experience.

In addition, the participants completed a questionnaire regarding their expectations and experience of the examination, reported in Busacchio et al. 2021 [17] that included a question on how informed they feel about the WB-MRI (from 1 = very poorly to 5 = very highly).

### 2.3. Statistical Analysis

The participants’ characteristics and previous history of screening tests were summarized by count and percent, while their ages were summarized by the mean and standard deviation (SD).

The main factors indicated by participants in the DT were categorized into clusters by an experienced psychologist according to the significant content.

Five clusters were identified: diagnostic certainty, psychological well-being, safety, test validity and time/costs. The values of each factor were summed up to produce the expected value for their specific cluster. The expected value for a cluster could range from –10 (very negative) to 0 (neutral) to +10 (very positive). The expected values for each cluster were cross-tabulated by procedure (Standard and WB-MRI+Standard).

The medians of the cancer risk perception (personal, comparative, and severity) distributions were used as cut-off values to dichotomize the participants for each aspect of risk perception. These were entered, together with the participants’ other characteristics, in a logistic regression analysis to estimate their association with the expected value attributed to the procedures for each of the five clusters. Positive cluster scores entered the logistic regression analysis as the event of interest.

Finally, between-cluster correlations and their contribution to the total explained variance as well as the dimensionality of the personality were explored by tetrachoric correlation (r) and Multiple Correspondence Analysis (MCA).

Results are presented as Odds Ratios (OR) with 95% Confidence Intervals (CI). Categorical variables were compared using the Fisher’s exact test or the one-sample test for proportions as appropriate, between procedures by the McNemar’s test. All tests were two-tailed, and considered significant at the 5% level. All analyses were carried out with SAS (version 9.4, Cary N.C., USA) with the exception of the MCA, which made use of R (version 3.6.3, R Core Team 2020, Vienna, Austria).

## 3. Results

### 3.1. Participant Characteristics

Seventy-six participants completed the questionnaires; the mean age was 54.6 (SD = 13.1) years, range 24 to 82 years. Forty-seven (61.8%) were male, 46 (60.5%) were educated to at least university level, and 58 (76.3%) never smoked. Of the 29 females, 24 (82.8%) reported a previous pap-test or mammography, while 32 (74.4%) out of 47 male participants had a previous PSA-test. Two (2. 6%) participants did not respond regarding prior faecal blood testing. Some 65 (85.5%) and 12 (21.1%) participants had a previous MRI or WBI-MRI, respectively (Table 1). The sample size was determined by thematic saturation (the point at which new data did not produce new knowledge).

### 3.2. Expected Value

For each self-generated factor in the DT, the participants indicated the subjective value he/she attributed to it (that is the subjective utility the procedure has for the participants in term of clinical and psychological consequences). More specifically, subjective values were scored from −10 (indicating an extreme negative consequence) to +10 (an extreme positive consequence), with zero indicating a no-relevance factor. We refer to negative scores as indicating negative expected values, and to positive scores as indicating positive expected values. A score of “0” was considered a neutral expected value. For each cluster, the main factors indicated by the participants were as follows:

(1) Diagnostic certainty: early diagnosis; scan of the whole body; detection of small and silent tumours.
(2) Psychological Well-being: lengthening life expectancy; greater personal well-being; increases serenity relative to health; serenity due to the effects of the examination; immediate discussion with the doctor; psychological well-being for one year.
(3) Safety: low biological risk of the test; no radiation exposure; repeatability; non-invasiveness; no contrast agent.
(4) Test validity: trust on and reliability of the institute; no need of further checks unless indicated; exploration of organs for which there is no prevention and for which screening is not foreseen; individuation of non-cancer diseases.
(5) Time/Cost: short-term exam and rapid outcome.

Overall, the participants expressed negative expected values regarding the standard procedure for all factor clusters (Table 2) with all attributing negative values for safety, text validity, cost/time (76; 100%), and the majority for diagnostic certainty (68; 89.5%); though only one participant (1; 1.3%) indicated a negative expected value for psychological well-being.

Regarding WB-MRI+Standard procedure, the majority of participants indicated a positive/very positive expected value regarding diagnostic certainty (N = 70; 92.1%) and safety (N = 56; 73.7%), but attributed a negative expected value to psychological well-being (N = 56, 73.7%), time/cost (N = 63; 82.9%) and test validity clusters (N = 60, 79.0%).

In the comparison between Standard vs WB-MRI+Standard procedures (Table 3), a significant association in favour of the WB-MRI+Standard procedure was seen for diagnostic certainty (*p* < 0.001) and for psychological well-being (*p* < 0.001), despite 42 (55.3%) patients evaluating both procedures neutral or negative.

### 3.3. Risk Perception and Personality Traits

For Personal risk, 49 (64.5%) of the participants gave values of at least 3, with a concomitant significant positive value for the WB-MRI+Standard procedure (*p* = 0.004). Their rating of comparative risk was also significantly associated with a higher positive expected value for the WB-MRI+Standard procedure (*p* = 0.04). Independently of the type of risk perception considered (personal vs comparative or severity), all participants associated a more negative expected value to the Standard procedure with a significant difference with respect to the 50% distribution proportion hypothesis for risk perception on the one-sample proportion test. There was no significant association between perception of cancer severity and the expected value attributed to the WB-MRI+Standard procedure (*p* = 0.79).

**Figure 1:**
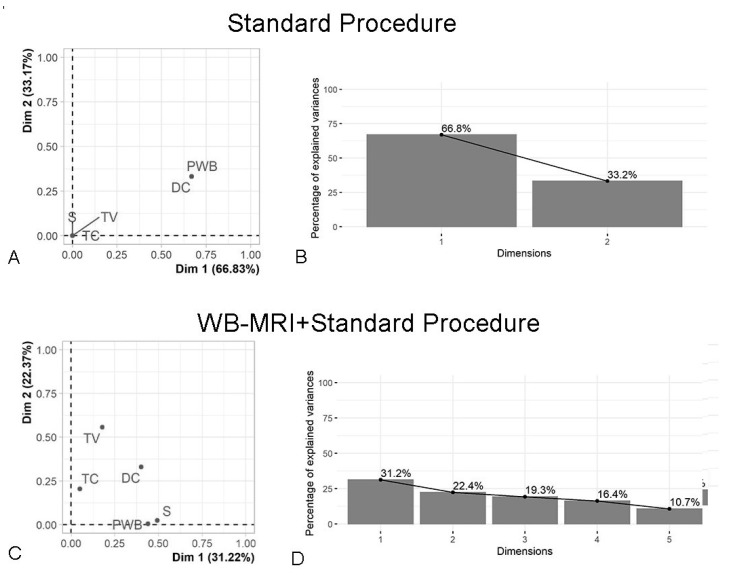
Illustration of the variability explained by the main factors (DC: Diagnostic Certainty; PWB: Psychological well-being; S: Safety; TC: Time/Cost; TV: Test validity). (**A**) For the Standard procedure, Multiple Correspondence Analysis showed diagnostic certainty and psychological well-being to account for the total explained variance, allowing the data to be described in two dimensions (**B**). (**C**) For the WB-MRI+Standard procedure, the variability involved five dimensions, with each dimension contributing similarly to the explained variance (**D**).

We then analysed which factors were the most informative for describing participants’ evaluation of options. For the Standard procedure, Multiple Correspondence Analysis showed a higher salience of diagnostic certainty and psychological well-being in evaluating the option: these two factors accounted for the total explained variance in two dimensions (Figure 1A,B). On the contrary, evaluation of the WB-MRI+Standard procedure required five dimensions with each cluster contributing similarly to the variance explained by the first two dimensions (Figure 1C,D). Diagnostic certainty was correlated with both the psychological well-being (r = 0.98) and the time/cost (r = 0.97). Other clusters were not correlated or were weakly correlated (minimum r = 0.02 between psychological well-being and time/cost, maximum r = 0.54 between diagnostic certainty and safety). Furthermore, no significant associations with expected value were found for the Standard and WB-MRI+Standard procedures. Finally, no association was found between options evaluated and personality factors measured with the BFI-10.

### 3.4. Level of Information about WBMRI

Prior to the WB-MRI examination, 44.6% of subjects felt they had a good to a very good level of information about the examination and 35% indicated a medium level of perceived information about the examination. Only 20% perceived to have a low level of information.

## 4. Discussion

In the present exploratory case-series study, we investigated the participants of the value attributed to the factors (advantages and disadvantages) associated with two options of cancer screening (standard procedure vs WB-MRI+Standard) and identified the main factors that individuals considered when they choose independently between the two options.

The main factors that participants considered were: diagnostic certainty, safety procedure and psychological well-being. Prior research has shown that patients generally prefer to wait less time, reduce the cancer risk due to radiation exposure, and undergo fewer scans with greater accuracy [29]. Bancroft et al., 2020 [30], indicated WB-MRI screening can be implemented in TP53 gene pathogenic variants and a previous cancer diagnosis may predict a better psychosocial outcome. The added value of the type of evaluation performed in the present study is to allow the participants to reflect on the two options simultaneously. Joint evaluation [31] reduces the focusing effect [32,33], relative to proposing only one option at a time as that generally induces people to not consider factors and options that are not made explicit during the judgment and decision-making process. Visualizing and reasoning on the expectations of the two options jointly allow creating a more complete and more precise mental representation of all the possibilities and possible consequences increasing participants’ awareness. Interestingly, the values attributed to the expected factors seemed to depend on personal and comparative cancer risk perception. In our study, participants with a higher personal and comparative (compared to the average women/men of the same age in Italy) cancer risk perception, perceived a higher expected value associated with the WB-MRI+Standard procedure and considered the standard procedure as providing lower utility. In fact, the perception that individuals have about a disease affects the health-protective actions performed to reduce their risks to get a disease and how they process and respond to new health information related to that diseases [34,35].

The results of the present work can help professionals understand the main factors individuals consider when approaching screening procedures and tailor communication according to the individuals’ risk perception tendency. The positive values expressed by subjects regarding WB-MRI for early cancer diagnosis indicated that MRI for this purpose seems to be acceptable to those motivated to pursue early cancer diagnosis. It is worth noting that WB-MRI is an adjunct to the standard procedure and not used in substitution. Therefore, the positive attitude expressed by subjects was related to the combination of examinations. In particular, the possibility to cover parts of the body not covered by standard procedures alone, together with the other main factors mentioned above seem to increase participants’ serenity.

Our study is subject to some limitations that have implications for the generalizability of the results. Firstly, only asymptomatic individuals who had self-referred for WB-MRI for early cancer diagnosis were included. This population is somewhat different from that likely to be encountered in a screening program and distinct from patients with a known disease. Nonetheless, our cohort represents a growing population that warrants consideration in light of the growing use of WB-MRI for early cancer diagnosis, both for high-risk individuals and amongst the worried well. Specific information that subjects had before the examination about the WB-MRI and perceptions of costs were not analysed. Almost (80%) of the participants did, however, indicated that they felt that they had a medium to a very good level of information about the examination, and all had accepted the cost of the examination. The type and balance of information held by subjects on WB-MRI need further investigation.

## 5. Conclusions

Considering that WB-MRI is an adjunct to the standard procedure and not a substitution, our results show the differences in individuals’ mental representation and value attribution about WB-MRI and standard cancer screening options in Italy. The greatest values attributed to WB-MRI were associated with diagnostic certainty, safe of procedure and psychological well-being. Risk perception has a significant role in determining the value attributed to either option, indeed the higher the perceived cancer risk the higher the value attributed to WB-MRI examination. In adopting WB-MRI as an examination with zero biological risks for early diagnosis in clinical practice, radiologists should implement a strategy of informing patients regarding the advantages and disadvantages related to the procedure as a means to reinforce the psychological well-being of the patients and their perception of the technique as reliable. Ad hoc decision tools, such as the DT used herein, can help physicians and patients improve awareness toward priorities and value and enhance confidence in the decision about health preventive behaviours. Future studies to measure awareness and identify means for enhancing patient confidence should be considered.

## Figures and Tables

**Table 1:** Patient’s characteristics, N = 76.

Characteristic	Level	Count (%)
Age, Mean (SD) Median	years	54.6 (13.1)
		54.0
Education, N (%)	Lower secondary school	6 (7.9%)
	High school	24 (31.6%)
	University degree	46 (60.5%)
Smoke, N (%)	Non-smoker	58 (76.3%)
	Ex-smoker	6 (7.9%)
	Smoker	12 (15.8%)
Sex, N (%)	Female	29 (38.2%)
	Male	47 (61.8%)
Previous exams, N (%)	Pap-test ^†^	24 (88.9%)
	Mammography ^†^	24 (88.9%)
	PSA-test ^‡^	32 (74.4%)
	Faecal blood test ^§^	45 (60.8%)
	MRI	65 (85.5%)
	WBI-MRI	12 (15.8%)

Note: ^†^ N = 27 female; ^‡^ N = 43 males only; ^§^ N = 74; SD = Standard Deviation; Percentages calculated on all valid cases.

**Table 2:** Early cancer diagnosis procedure scores expected value ^†^ distribution by cluster, N = 76.

		Procedure, N (%)	
Cluster	Score	Standard	WB-MRI+Standard Procedure
Diagnostic Certainty	≤0	68 (89.5)	6 (7.9)
	>0	8 (10.5)	70 (92.1)
Psychological well-being	≤0	75 (98.7)	42 (55.3)
	>0	1 (1.3)	34 (44.7)
Safety	≤0	76 (100)	20 (26.3)
	>0	0	56 (73.7)
Test validity	≤0	76 (100)	60 (79.0)
	>0	0	16 (21.0)
Time/Cost	≤0	76 (100)	63 (82.9)
	>0	0	13 (17.1)

^†^ Expected value Scores: Score ≤ negative expected values 0 = Neutral or less expected value; Score > 0 = positive expected value.

**Table 3:** Comparison of participant value attribution factor clusters between diagnostic procedures (Standard Procedure or WB-MRI+Standard Procedure), N = 76.

		WB-MRI+Standard Procedure N (%)		
Cluster	Standard Procedure	≤0	>0	*p*-Value
Diagnostic Certainty	≤0	6 (7.9)	62 (81.6)	
	>0	0	8 (10.5)	<0.001
Psychological well-being	≤0	42 (55.3)	33 (43.4)	
	>0	0	1 (1.3)	<0.001
Safety	≤0	20 (26.3)	56 (73.7)	
	>0	0	0	-
Test validity	≤0	60 (78.9)	16 (21.1)	
	>0	0	0	-
Time/Cost	≤0	63 (82.9)	13 (17.1)	
	>0	0	0	-

Value Score: Neutral or negative value, Score ≤ 0; positive value, Score > 0; b McNemar Test.

## Data Availability

The data is available for review on request to the corresponding author.
